# A Flexible Membrane May Improve Bone Regeneration by Increasing Hydrophilicity and Conformability in Lateral Bone Augmentation

**DOI:** 10.34133/bmr.0113

**Published:** 2024-11-18

**Authors:** Dongseob Lee, Young-Chang Ko, Ki-Tae Koo, Yang-Jo Seol, Yong-Moo Lee, Jungwon Lee

**Affiliations:** ^1^ Department of Periodontology, School of Dentistry and Dental Research Institute, Seoul National University, Seoul, Korea.; ^2^ National Dental Care Center for Persons with Special Needs, Seoul National University Dental Hospital, Seoul, Korea.; ^3^ One-Stop Specialty Center, Seoul National University Dental Hospital, Seoul, Korea.

## Abstract

Collagen membranes play a crucial role in guided bone regeneration (GBR) by preventing soft tissue infiltration and maintaining space for bone formation. This study investigated the impact of collagen membrane flexibility on GBR outcomes through in vitro and in vivo analyses. Flexible (0.3 mm in width) and stiff (0.5 mm in width) porcine collagen membranes were compared. In vitro tests assessed hydrophilicity, enzymatic degradation, conformability, space maintenance, and tensile strength. An in vivo study using a canine model evaluated bone regeneration in standardized mandibular defects filled with deproteinized porcine bone mineral and covered with no membrane, flexible membrane, or stiff membrane. Micro-computed tomography and histomorphometric analyses were performed at 8 and 16 weeks. The flexible membrane demonstrated superior hydrophilicity, faster enzymatic degradation, and greater conformability in vitro. In vivo, micro-computed tomography analysis revealed similar alveolar ridge widths across all groups. Histomorphometric analysis at 16 weeks showed significantly larger regenerated areas in the flexible membrane group compared to controls in coronal, middle, and apical regions. Both membrane groups exhibited higher regeneration ratios than controls, with significant differences in the coronal area. The flexible membrane group demonstrated significantly higher new bone formation in all regions compared to controls at 16 weeks. These findings suggest that flexible membrane substantially enhances GBR outcomes by increasing hydrophilicity and conformability. The study highlights the potential clinical benefits of incorporating flexible membranes in GBR procedures for improved bone regeneration outcomes.

## Introduction

The barrier membrane plays a crucial role in guided bone regeneration (GBR) by preventing the infiltration of epithelial cells and enhancing space maintenance, which facilitates the migration of osteogenic cells during bone regeneration [1,2]. Due to the importance of the barrier membrane, numerous studies have focused on its mechanical properties and application methods [3–5].

Considering the original function of the membrane, which is to suppress the ingrowth of epithelial cells and stabilize bone substitutes, enhancing the physical properties like tensile strength and elasticity and/or flexibility of the collagen membrane could potentially affect bone regeneration. However, in a preclinical study, membrane that demonstrated higher tensile strength was not shown significance in new bone areas (%) with radiographic and histologic analysis [6]. Flexible membrane demonstrated better occlusion effect and accelerated bone regeneration [7]. These findings suggest that the membrane’s flexibility and/or elasticity might influence the result of bone regeneration rather than its tensile strength. According to the review paper, elasticity and/or flexibility per se does not appear to affect the outcome of bone regeneration [1]. The appropriate elasticity of the membrane might provide for maintaining the space needed for bone substitutes leading to good regeneration outcomes [8,9]. However, the impact of membrane elasticity on bone regeneration outcomes has not been thoroughly investigated.

According to a recent systematic review, descriptive statistics indicate that GBR using block bone grafts results in greater bone augmentation compared to particulate bone substitutes, although this was not a direct comparison between the 2 groups [10]. Another study demonstrated that block bone grafts had been shown histologically better results than particulate bone substitutes [11]. The improved outcomes with block bone grafts may be attributed to better space maintenance during the bone regeneration period. However, it is widely acknowledged that there is some reluctance to use autogenous block bone grafts in clinical settings due to increased patient morbidity like swelling and the potential for various surgical complications [12].

In the context of bone regeneration, various techniques have been employed to maintain space, including the use of screws, titanium-reinforced nonresorbable membranes, or titanium mesh [13–15]. However, these methods require the subsequent removal of the screw or membrane, which can be inconvenient. Recently, to circumvent these drawbacks, several bone grafting techniques utilizing collagen soft block bone substitutes with collagen membrane have been developed, with encouraging clinical outcomes reported [16,17]. Collagen membrane might be considered convenient to use comparing titanium-reinforced nonresorbable membranes or titanium. Because there is no need for additional surgery to harvest block bone from donor site, using soft block bone substitutes for horizontal bone augmentation may reduce clinical inconvenience and patient morbidity like swelling comparing autogenous block bone [12].

Studies have investigated the primary stability of bone-augmented sites using various combinations of bone substitutes during wound closure [18,19] Particulate bone substitutes tend to displace during suturing procedures, resulting in a greater reduction of horizontal bone width compared to block-type bone substitutes. However, when extensive horizontal bone augmentation is needed, the size of available block-type bone substitutes is limited. In such cases, it is necessary to use them in conjunction with particulate bone substitutes in clinical situations. Specifically, a technique can be employed where block-type bone substitutes are applied to the apical area to support the particulate bone substitutes placed in the upper area.

This study aimed to compare flexible collagen membrane with stiff collagen membranes by in vitro, as well as through radiographic and histomorphometric analyses in vivo.

## Materials and Methods

### Collagen membrane

**Fig. 1.**
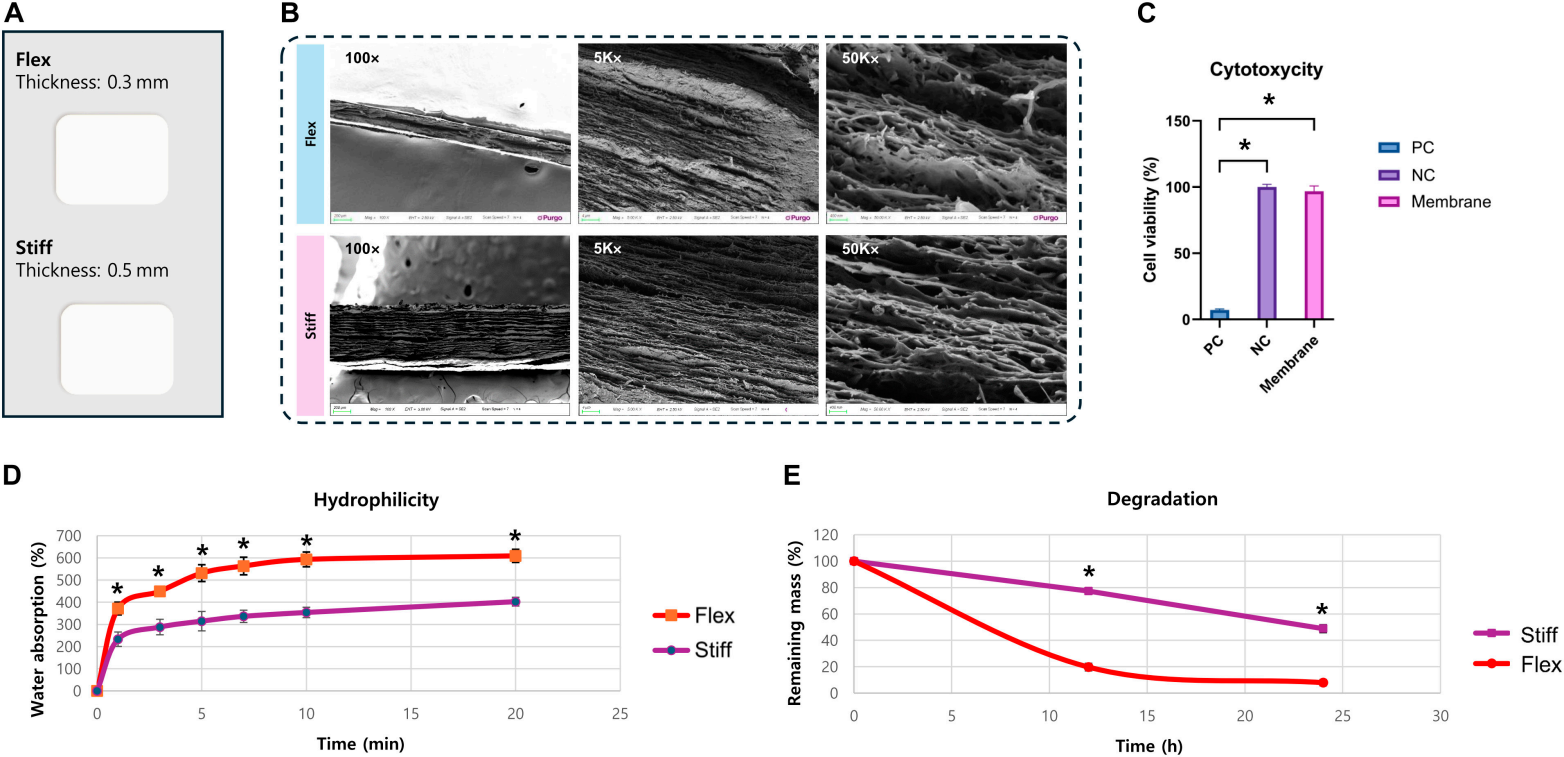
Characterization of the flexible and stiff collagen membranes. (A) Schematic representation of the flexible and stiff collagen membranes. (B) FE-SEM images of flexible and stiff collagen membranes surface and cross-section. FE-SEM, field emission scanning electron microscopy. (C) Cytotoxicity assay results show high cell viability of Balb/c 3T3 cells cultured on the collagen membrane, comparable to the PC and NC. The data indicate high biocompatibility of the collagen membrane. The Kruskal–Wallis test with post-hoc test was performed for statistical analysis (*n* = 6) (^*^P < 0.05). PC, positive control; NC, negative control. (D) Hydrophilicity assessment of flexible and Stiff collagen membranes. The Mann–Whitney *U* test was performed for statistical analysis (*n* = 4) (^*^P < 0.05). (E) Enzymatic stability results of flexible and stiff membranes. The Mann–Whitney *U* test was performed for statistical analysis (*n* = 3) (^*^P < 0.05).

The flexible and stiff collagen membranes are made from natural porcine fibrous type I collagen. Two types of membranes were utilized: cross-linked collagen membranes with a thickness of 0.3 mm (flexible membrane) and cross-linked collagen membranes with a thickness of 0.5 mm (stiff membrane) (Fig. 1A). All other manufacturing processes remained the same.

### Surface characteristics

The collagen samples were uniformly coated with platinum using an ion sputtering technique (Ion sputter coater, GSEM Co., Ltd., G20, Korea) to enhance electrical conductivity. The surface and cross-sectional morphology of the samples were then examined using field emission scanning electron microscopy (FE-SEM, Carl Zeiss, Sigma 300, Germany).

### In vitro cytotoxicity and biocompatibility

Stiff membrane was assessed for its potential to induce cytotoxicity in Balb/c 3T3 cells using the elution method. Since the 2 membranes differed only in thickness and flexibility, the stiffer membrane was selected for the cytotoxicity test. The test item was extracted at a ratio of 6 cm^2^/ml (given that the thickness of the test item was less than 0.5 mm) in 1× Dulbecco’s Modified Eagle’s Medium (DMEM) supplemented with 5% heat-inactivated newborn calf serum and 1% penicillin/streptomycin solution (extraction medium) at 37 ± 1 °C for 72 h. It was found that one entire test item could absorb 2.9 ml of extraction medium. A test item with an area of approximately 74.1 cm^2^ (3 units) was extracted in 21.1 ml (extraction volume − 12.4 ml + absorption volume − 8.7 ml) of extraction medium at 37 ± 1 °C for 72 h, under aseptic conditions. A sterilized high-density polyethylene film (negative control), measuring 18 cm^2^ (both sides), was extracted at a ratio of 3 cm^2^/ml of solvent in 6 ml of extraction medium at 37 ± 1 °C for 72 h. The positive control, sodium lauryl sulfate, was freshly prepared (before treatment of the cells) at a final concentration of 0.15 mg/ml. This meets the requirements of ISO 10993-12:2012(E), ISO 10993-12:2021(E), and ISO 10993-5:2009(E). At the end of the extraction period, the extract was clear, and no color change or particulates were observed. The retrieved test item appeared swollen. Therefore, no additional processing such as filtration, centrifugation, pH adjustments, or any other processing was performed. Extracts were used within 36 min and were considered stable during this period. Exponentially growing Balb/c 3T3 cells were seeded in a 96-well plate at a concentration of 1 × 10^4^ cells/well. After 24 h of incubation, the cells reached approximately 80% confluence. The complete growth medium was then removed from all wells, and 6 replicates of the test item extract (100%) and neat extract of the negative control (100%) were added to their respective culture wells. The plate was incubated at 37 °C with 5% CO_2_ for 24 h. After this incubation period, the cells were evaluated both qualitatively (microscopic evaluation) to assess cell morphology and quantitatively (neutral red uptake method [20]) to determine cell viability (*n* = 6).

### Hydrophilicity

The weight of the material was measured prior to hydration. It was subsequently immersed in a beaker filled with normal saline. Samples were collected at specified time intervals—1, 3, 5, 7, 10, and 20 min after hydration—and their weight was determined using a quantitative scale (*n* = 4) [21].

### Enzymatic stability

The enzymatic stability of the 2 membranes was assessed using an in vitro collagenase resistance test [22]. For each product, collagen membranes were prepared with dimensions of 15 × 20 mm. Prior to testing, all samples and enzyme solutions were incubated separately at 37°C. The evaluation involved incubating the samples at 37°C in 15 ml of a 5 U/ml collagenase solution from *Clostridium histolyticum* (Sigma, 50 mM Tris-HCl buffer and 0.36 mM CaCl_2_, pH 7.6). Samples were collected and dried at 12- and 24-h time points, and their weights were measured using a quantitative scale (*n* = 3).

### Conformability

**Fig. 2.**
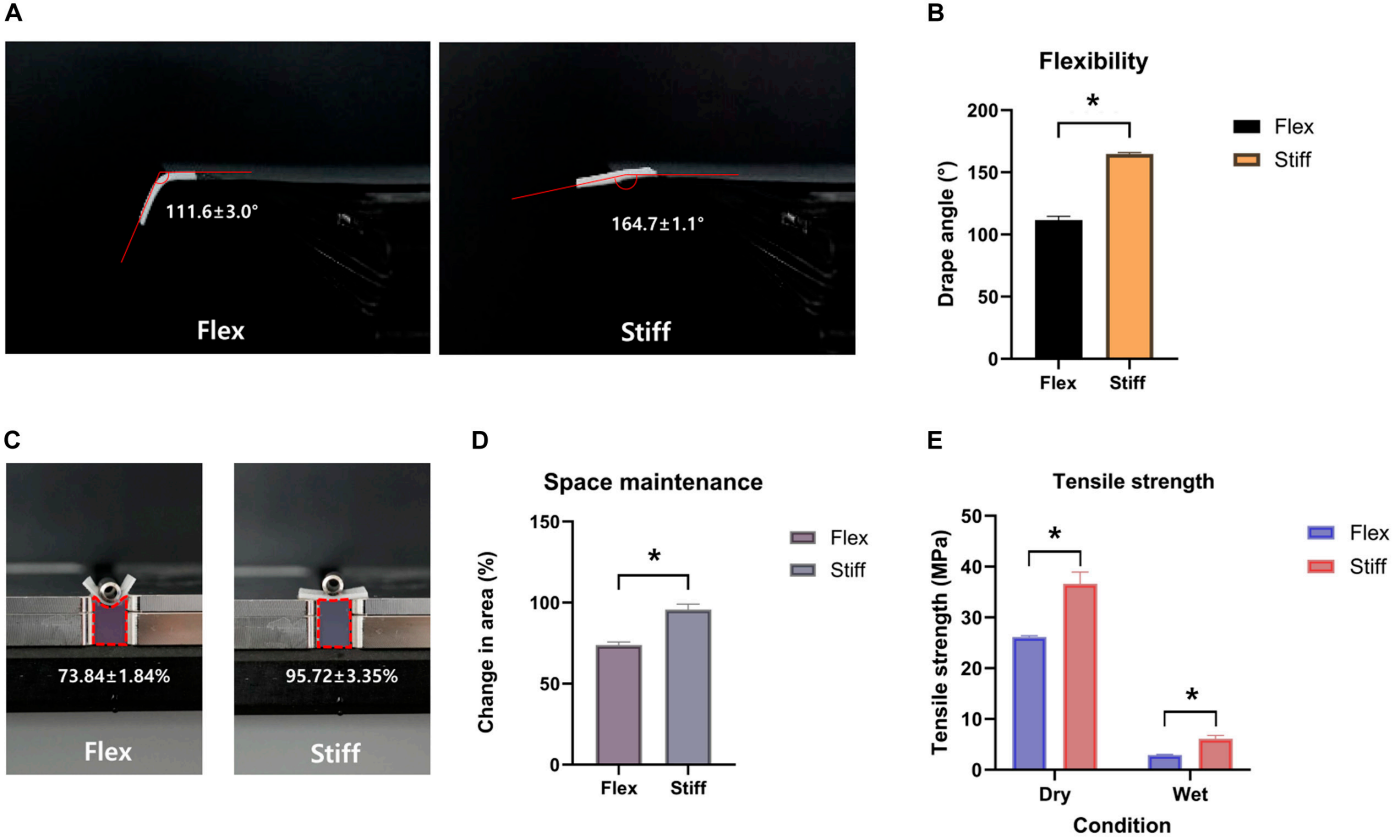
Evaluation of conformability, space maintenance, and tensile strength of flexible and stiff collagen membranes. (A) Schematic representation of the drapability test used to measure conformability for 2 types of membranes. (B) Conformability results showing the drape angles of flexible and stiff membranes. The flexible membrane demonstrated a significantly smaller bending angle (111.6° ± 3.0°) compared to the stiff membrane (164.7° ± 1.1°). The Mann–Whitney *U* test was performed for statistical analysis (*n* = 6) (^*^P < 0.05). (C) Illustration of the space maintenance test where hydrated flexible and stiff membranes were subjected to a load of 3.32 g. (D) Comparative results of space maintenance between flexible and stiff membranes. The stiff membrane maintained a significantly larger area (95.72% ± 3.35%) than the flexible membrane (73.84% ± 1.84%) under the same load. The Mann–Whitney *U* test was performed for statistical analysis (*n* = 3) (^*^P < 0.05). (E) Tensile strength measurement results of the flexible and stiff membranes under dry and wet conditions. The Mann–Whitney *U* test was performed in dry or wet condition, respectively, for statistical analysis (*n* = 3) (^*^P < 0.05).

Conformability was assessed using the conformability test as outlined in ISO 9073-9 Textiles—Test methods for the Drapability of Fabrics. Samples measuring 2 × 3 cm were initially hydrated with water for 2 min (Fig. 2A). After removing excess surface water, the hydrated samples were positioned on a rectangular block, allowing half of each membrane’s length to drape over the block’s edge. Subsequently, the angle of the draped samples was measured (*n* = 6). The conformability grade was categorized as follows: 90° to 115° (complete), 115° to 140° (high), 140° to 165° (moderate), and 165° to 180° (minimal).

### Space maintenance

The flexible and stiff membranes were adequately hydrated. Once the hydrated products were in place, a load of 3.32 g was applied (Fig. 2C). The change in the area beneath the membrane before and after applying the load was analyzed using ImageJ (*n* = 3).

### Tensile strength

The mechanical properties of flexible and stiff membranes were evaluated according to the international standard ASTM 2150-19. Each sample was assessed using a Universal Testing Machine (UTM, TO-101, Test-one, Korea) set to a force of 10 kgf/mm^2^ and a crosshead speed of 50 mm/min. Membrane specimens were prepared in dimensions of 15 mm in width and 20 mm in length. These specimens underwent testing in both dry and wet states, the latter following a 5-min immersion in distilled water. The tensile strength of the membranes was determined by calculating the average maximum tensile strength for each sample (*n* = 3).

### Experimental animals and sample size estimation

The experiment protocol was approved by the Institutional Animal Care and Use Committee of Seoul National University (approval no. SNU-230306-4-1). The manuscript was written following the ARRIVE 2.0 guideline.

Six male beagle dogs, each 1-year-old and weighing between 10 and 13 kg, were included in the study. All animals were both systemically and periodontally healthy at the time of recruitment. They were acclimated to the facility for 2 weeks prior to the experiment. Each dog was housed in an individual indoor kennel measuring 90 cm in width, 80 cm in depth, and 80 cm in height. The animals had free access to water and were fed a standard pellet dog food diet.

The sample size was estimated using G*power (version 3.1.9.7, Autenzell, Germany). A previous study showed that the use of bone substitute materials with a membrane (47%) resulted in a higher mineralized structure compared to the group that used only a membrane (37%) [23]. The sample size was ultimately calculated to be 6 when the standard deviation and mean differences were both set at 10%, with an alpha (α) of 5% and a power (1 − β) of 80%.

### Timeline and surgical procedures

**Fig. 3.**
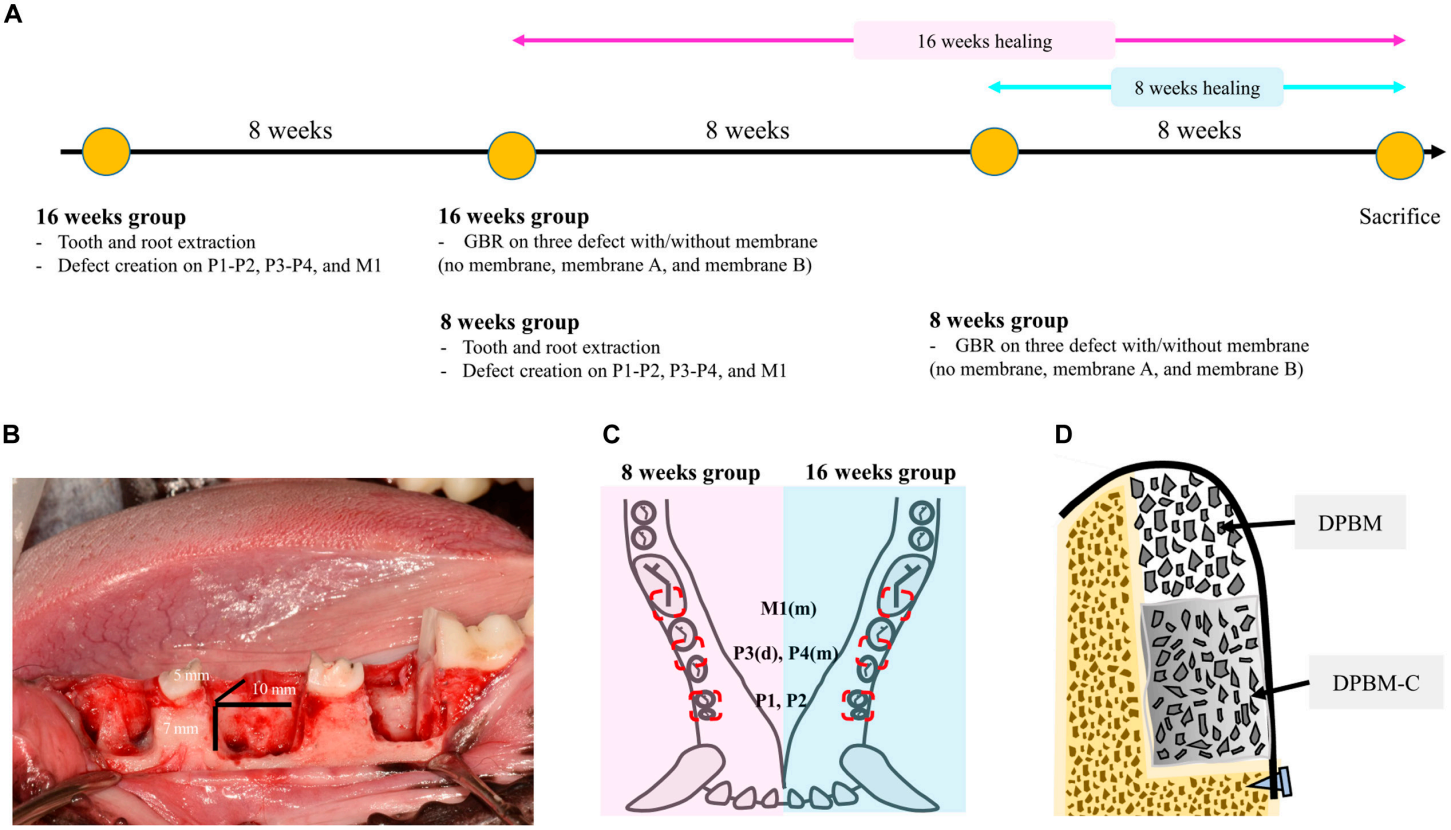
Timeline and schematic diagram of the experiment. (A) Timeline of this experimental study. (B) Defect configuration. (C) Allocation of experimental sites. (D) Schematic diagram of guided bone regeneration in this study.

A timeline of the animal experiment design is illustrated in Fig. 3A. The lower hemimandible premolars and the first molar (lower first and second premolars: P1, P2; hemi-sectioned distal root of the third premolar: P3(d); and both hemi-sectioned mesial roots of the fourth premolar: P4(m) and first molar: M1(m)) were extracted. Following the endodontic treatment of the remaining mesial root of the third premolar, the distal root of the fourth premolar, and the distal root of the first molar, box-shaped defects were created at the P1–P2, P3(m)–P4(d), and M1(m) sites using a rotary instrument with burs. The standardized defects were box-shaped, measuring 10 mm mesiodistally, 5 mm buccolingually, and 7 mm apicocoronally (Fig. 3B). After an 8-week healing period at the defect site, a GBR procedure was performed using 2 types of bone substitutes: collagenated deproteinized porcine block bone mineral (DPBM-C, Lego Graft, Purgo, Sungnam, Gyeonggi-do, Korea) and deproteinized porcine bone mineral (DPBM, THE graft-cortical, Purgo, Sungnam, Gyeonggi-do, Korea). One of 3 options—no membrane, flexible membrane (The cover flex, 25 × 30 mm, 0.3 mm thickness, Purgo, Sungnam, Gyeonggi-do, Korea), or stiff membrane (The cover stiff, 25 × 30 mm, 0.5 mm thickness, Purgo, Sungnam, Gyeonggi-do, Korea)—was randomly applied to the graft site (Fig. 3C). All membranes were secured with a titanium pin (Bone tack, Osstem, Seoul, Korea) (Fig. 3D).

• Control: bone substitutes
• Flex: bone substitutes + flexible membrane
• Stiff: bone substitutes + stiff membrane

**Fig. 4.**
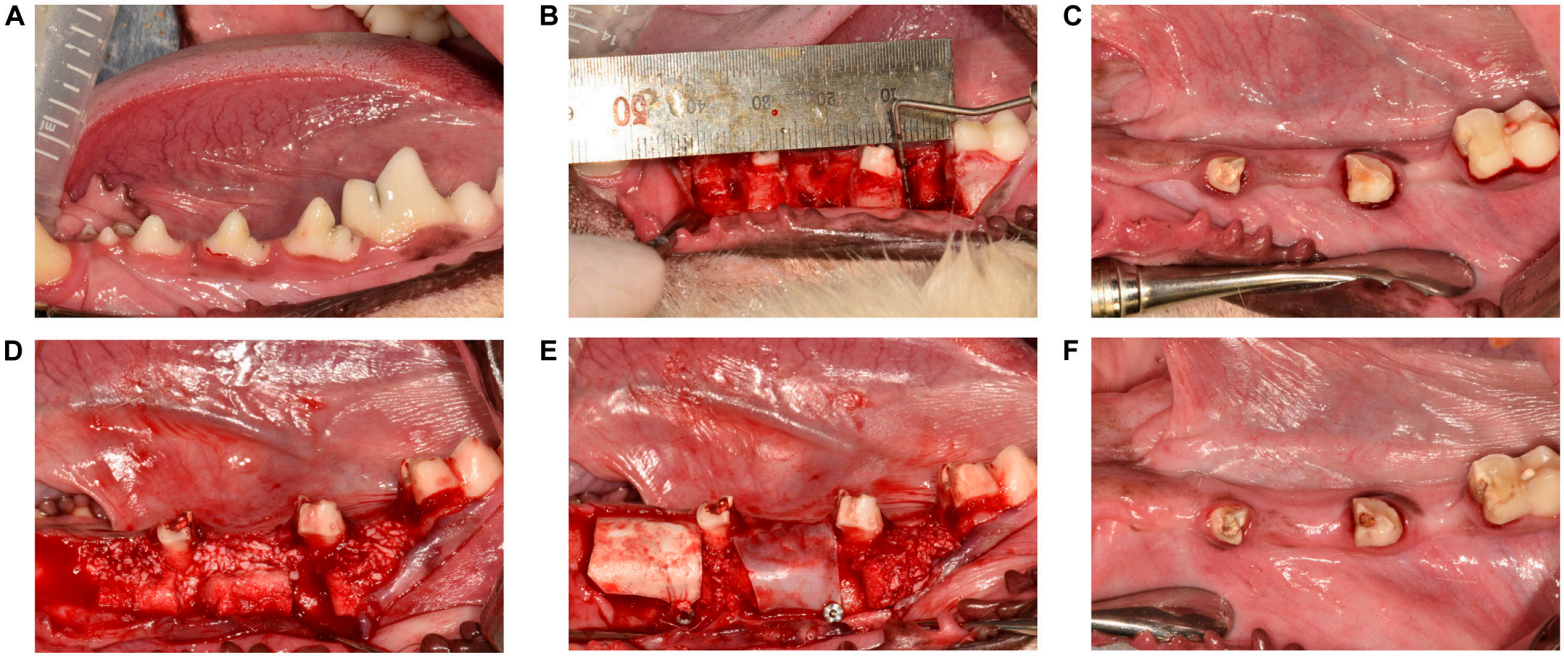
Clinical photos of surgical procedures. (A) Clinical photo before teeth extraction and defect creation procedure. (B) The extraction of the lower first premolar (P1) and second premolar (P2), hemi-section and extraction of the distal root of the third premolar (P3(d)), the mesial roots of the fourth premolar (P4(m)), and the mesial roots of the first molar (M1(m)). Box-shaped defect creation at the P1–P2, P3(m)–P4(d), and M1(m) sites measuring 10 mm mesiodistally, 5 mm buccolingually, and 7 mm apicocoronally. (C) Eight weeks healing period after defect creation. (D) Bone substitute augmentation at each experimental sites divided into 3 groups: non-membrane, flexible membrane, or stiff membrane group. (E) Covering each membrane with bone tack. (F) Eight weeks postoperation photo of bone augmentation.

Each hemimandible was allowed to heal for either 8 or 16 weeks, respectively. All surgical procedures are illustrated in Fig. 4A to F.

### Animal care and euthanasia

After the operation, the animals received an intravenous administration of 20 mg/kg antibiotics (cefazolin; Chongkundang Pharmaceutical Corp, Seoul, Korea) and analgesics (Toranzin 5 mg/kg; Samsung Pharm., Gyeonggi-do, Korea). Additionally, antibiotics (amoxicillin 500 mg; Chongkundang Pharm., Seoul, Korea) and analgesics (ibuprofen 400 mg; Daewoong Pharm., Seoul, Korea) were mixed into the animals’ diet for 3 days after surgery. All stitches were removed 14 days after surgery.

The animals were euthanized following 8- and 16-week bony healing periods via carotid injection using a lethal dose of potassium chloride (7 5mg/kg; Jeil Pharm., Daegu, Korea). Block biopsies encompassing the experimental sites were then collected for radiographic and histologic analyses. The harvested hemimandibles were rinsed in saline and fixed in 10% buffered formalin.

### Micro-computed tomography scanning

Before histological preparation, micro-computed tomography (micro-CT) data of the samples were obtained under the same conditions as in previous studies [24–26] (Fig. S1A to C). Radiographic analysis was conducted using CTAn software (Bruker-CT, Kontich, Belgium) and ImageJ software (National Institutes of Health, Bethesda, MD, USA). A linear analysis was performed to measure the overall width, including pristine bone, new bone, and graft materials at each augmented site in the buccolingual direction. Linear measurements were taken in 1-mm increments from 1 to 6 mm apical of the alveolar crest in the apical direction (Fig. S1D to I).

The volume of new bone, residual bone graft particles, and total bone within the volume of interest was measured using distinct 8-bit grayscale thresholds, set at 45 to 69 for new bone and 70 to 255 for residual bone substitutes, as determined by a previous study [24]. Within the specified range for new bone, several parameters were assessed: total volume (TV), bone volume/tissue volume (BV/TV), bone surface/bone volume (BS/BV), trabecular bone pattern factor (Tb.Pf), structure model index (SMI) value, trabecular thickness (Tb.Th), trabecular number (Tb.N), and trabecular separation (Tb.Sp).

### Histologic and histomorphometric analysis

All specimens were dehydrated in an ascending series of alcohol concentrations and then embedded in methylmethacrylate resin blocks. Each block was sectioned using a diamond cutter (Exakt, Norderstedt, Germany). The center of each GBR site was sawed in the buccolingual direction to prepare histologic sections. The specimens were initially sectioned to approximately 100-μm thickness and subsequently ground down to 30 μm using a diamond grinder. All slides were stained with Picrosirius Red Stain. Digital images of all histologic slides were obtained through scanning. Histological analysis was conducted using Photoshop 2024 (Adobe Systems, San Jose, CA, USA) and ImageJ (National Institutes of Health, Bethesda, MD, USA).

**Fig. 5.**
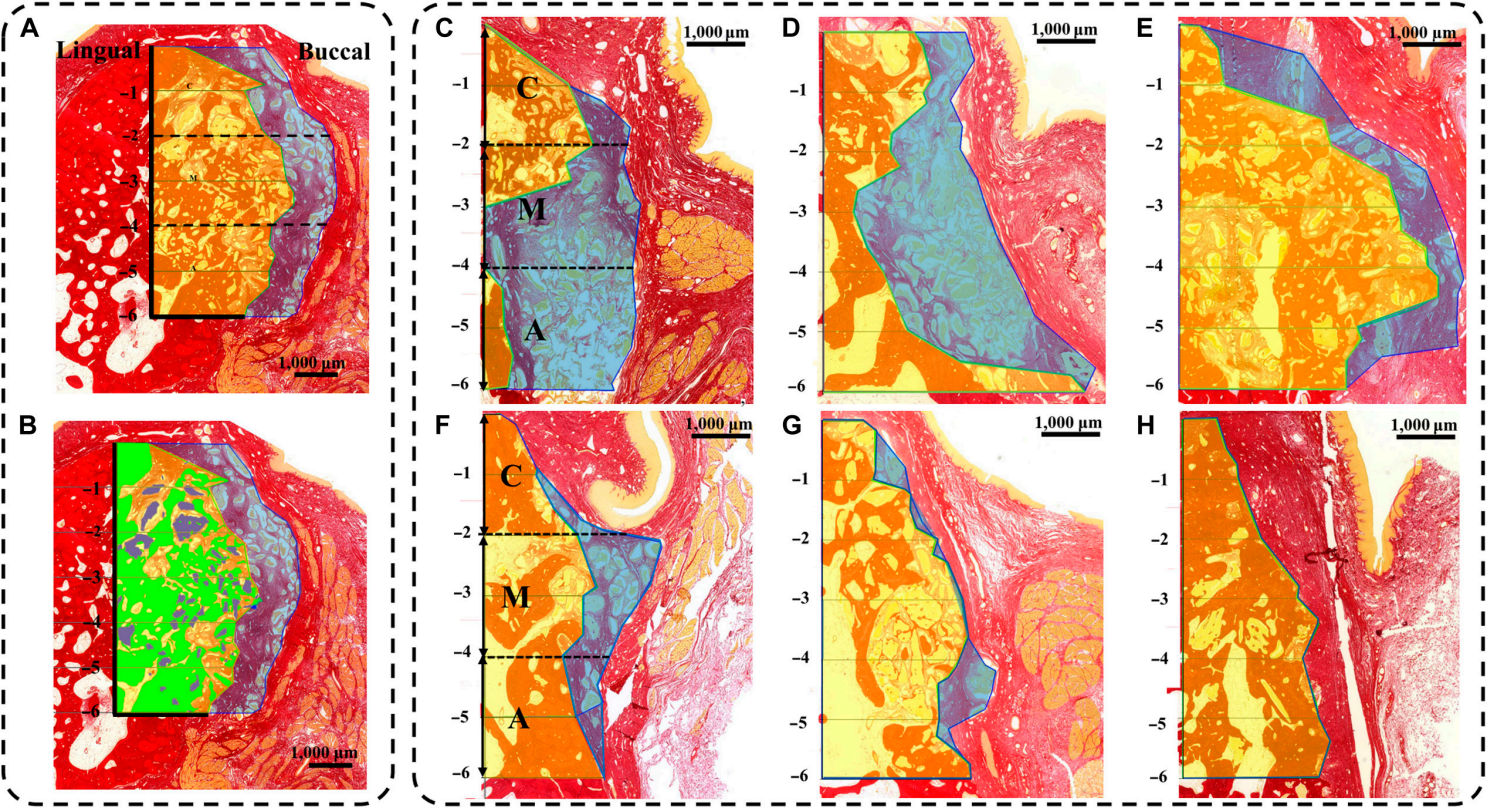
The representative pictures for regenerative area with 3 components and augmented area. (A) The regenerated area (painted yellow) and the augmented area (painted yellow and blue) are demarcated by the coronal (C), middle (M), and apical (A) areas, each divided into 2-mm intervals. (B) The colored components of the regenerated area include new bone (NB), depicted in green; residual bone particle (RBP), in blue; and fibrovascular connective tissue (FVCT), in red. (C to E) Representative figures of the control, flex group, and stiff group, respectively, at 8 weeks of healing. (F to H) Representative figures of the control, flex group, and stiff group, respectively, at 16 weeks of healing.

On histologic images, a vertical line distinguishing pristine bone from newly formed bone after defect creation was identified and marked. Based on vertical location, 3 regions were delineated and labeled: the coronal area, spanning from 0 to 2 mm apical of the alveolar crest; the middle area, from 2 to 4 mm apical of the alveolar crest; and the apical area, from 4 to 6 mm apical of the alveolar crest. The regenerated and augmented areas in each region were detected and quantified using histologic images (Fig. 5A and C to H). The regeneration ratio was also calculated and compared across groups.

• Regenerated area (mm^2^): the area where newly formed bone was observed as the mineralized area near preexisting lamellar bone from the lingual and apical sides, including mineralized tissue, residual bone material, and fibrovascular connective tissue (FVCT).
• Augmented area (mm^2^): the area surrounded by the regenerated area and the periosteum-like dense connective tissue layer from the buccal side.
• $$\text{Regeneration ratio}\;\left(\%\right)=\frac{\text{Regenerated area}}{\text{Augmented area}}\times 100$$

Three components of the regenerated area were measured, as shown in Fig. 5B: new bone (NB), depicted in green; residual bone particle (RBP), shown in blue; and FVCT, represented in red. The percentage of NB per regenerated area (new bone percent) was also calculated.

### Statistical analysis

Values are presented as means ± standard deviations and median [min, max]. The Mann–Whitney *U* test was performed to compare 2 groups and the Kruskal–Wallis test was used to compare 3 groups with the Bonferroni correction for the post-hoc test using SPSS (version 25.0, IBM Corp., Armonk, NY, USA) and GraphPad software (version 10.0.2, San Diego, CA, USA). Statistical significance was established at *P* < 0.05. Intraclass correlation coefficients (ICCs) were calculated with repeated histological and radiological measurements using 2-way mixed effect model. The ICC value was 0.904 for histological measurement and 0.952 for radiological measurement, respectively (95% confidence interval). Measurements were conducted 5 times at 1-week intervals to assess intra-examiner reliability.

## Results

### Surface texture and cross-sectional features

The surface characteristics of the membrane are depicted in Fig. 1B. At ×5,000 magnification, the collagen membrane exhibited a relatively smooth texture with minor depressions, indicating a uniform distribution of collagen fibers. At ×50,000 magnification, the surface displayed a more detailed network of collagen fibrils. These fibrils are densely packed, forming a compact and interconnected structure.

A cross-sectional view at ×100 magnification revealed a multilayered structure with distinct layers of collagen fibers, with each layer appearing well-defined and highly organized. At ×5,000 magnification, the cross-sectional image emphasizes the alignment of collagen fibers, which are parallel and tightly packed. At ×50,000 magnification, the detailed fibrillar structure of the collagen membrane became apparent. The collagen fibers displayed a fine, interconnected network, essential for inhibiting the ingrowth of epithelial cells.

### Cell viability

The cell viability assay revealed that cells cultured on the collagen membrane exhibited high viability, comparable to that of the positive control. This indicates that the cross-linked collagen membrane possesses high biocompatibility (Fig. 1C).

### Water absorbability

The time-dependent comparison of water absorption levels revealed that the flexible membrane generally exhibited higher water absorption than the stiff membrane (Fig. 1D). Significant differences between the 2 groups were observed at each time point (*P* < 0.05), indicating that the flexible membrane had a superior water absorption rate than the stiff membrane.

### Degradation resistance

The time-dependent comparison of the remaining material revealed that the group treated with the flexible membrane generally exhibited a faster decline in the rate of remaining mass than the group where the stiff membrane was used (Fig. 1E). Significantly lower remaining mass was observed in the flexible membrane group than in the stiff membrane group at the 12- and 24-h time points (*P* < 0.05). These results demonstrate that the flexible membrane group had a higher degradation rate than the stiff membrane group.

### Bending angle

The flexible membrane exhibited a significantly smaller bending angle than the stiff membrane (111.6° ± 3.0° vs. 164.7° ± 1.1°, respectively) (Fig. 2B). Based on the drape angle, the flexible membrane group was classified as “completely” conformable and the stiff membrane group was classified as “moderately” conformable.

### Space maintenance

Figure 2D illustrates the comparative results of space maintenance between the flexible and stiff membranes. The data show a statistically significant difference between the 2 groups, with the stiff membrane maintaining a larger area compared to the flexible membrane under the same load (95.72% ± 3.35% vs. 73.84% ± 1.84%, respectively).

### Mechanical property

The results of the tensile strength tests for the flexible and stiff membranes under both wet and dry conditions are presented in Fig. 2E. The stiff membrane demonstrated significantly higher tensile strength than the flexible membrane in both conditions. Furthermore, the tensile strength was generally greater in the dry condition for both types of membranes.

### Clinical observation

During the healing periods, no specific signs of inflammation were observed at any of the experimental sites (Fig. S2).

### Radiographic analysis

In sites with defects, bone filling with graft materials was observed after GBR, as illustrated in Fig. S1A to C. Volumetric analysis showed no significant differences in BV/TV, BS/BV, Tb.Pf, SMI, Tb.Th, Tb.N, and Tb.Sp among the groups at each healing time point, as detailed in Table S1. Similarly, the overall width at each vertical depth showed no statistical differences among the groups at each time point, as presented in Table 1.

**Table 1.** Width measurements from bone to graft materials at each vertical height on micro-CT images. Values are presented as mean ± standard deviation and median [min, max]. The Kruskal–Wallis test was performed for statistical analysis (^*^P < 0.05). Control: bone substitutes. Flex group: bone substitutes + flexible collagen membrane. Stiff group: bone substitutes + stiff collagen membrane.

Depth	Control (mm)	Flex group (mm)	Stiff group (mm)	*P* value
8 weeks				
−1 mm	5.6 ± 0.9	5.3 ± 0.9	4.9 ± 0.8	0.42
	5.6 [4.1, 6.6]	5.2 [4.2, 6.8]	4.9 [4.1, 5.8]	
−2 mm	6.6 ± 1.4	5.9 ± 1.0	5.8 ± 1.1	0.35
	7.0 [3.9, 7.9]	5.7 [4.8, 7.8]	6.4 [4.6, 7.4]	
−3 mm	7.2 ± 1.2	6.6 ± 0.8	6.6 ± 1.3	0.42
	7.3 [5.3, 8.8]	6.4 [5.8, 8.1]	6.3 [5.2, 8.3]	
−4 mm	7.8 ± 0.9	7.0 ± 0.7	6.9 ± 1.4	0.41
	7.8 [6.7, 9.0]	6.9 [6.1, 8.3]	6.8 [5.3, 9.0]	
−5 mm	8.2 ± 0.6	7.2 ± 0.6	7.3 ± 1.4	0.13
	8.1 [7.4, 9.0]	7.3 [6.5, 8.1]	7.6 [5.2, 9.3]	
−6 mm	8.0 ± 0.4	7.6 ± 0.4	7.8 ± 1.2	0.61
	7.9 [7.5, 8.6]	7.8 [7.1, 8.0]	7.9 [6.4, 9.7]	
16 weeks				
−1 mm	3.4 ± 0.8	4.5 ± 0.3	4.2 ± 0.6	0.06
	3.6 [2.5, 4.5]	4.4 [4.1, 5.0]	4.1 [3.4, 5.3]	
−2 mm	5.1 ± 1.0	5.5 ± 0.6	5.2 ± 0.6	0.82
	5.2 [3.6, 6.7]	5.3 [4.9, 6.7]	5.3 [4.5, 6.3]	
−3 mm	5.6 ± 1.0	5.8 ± 0.9	5.9 ± 1.0	0.91
	5.6 [4.5, 7.3]	5.4 [5.0, 7.5]	5.7 [4.5, 7.3]	
−4 mm	5.9 ± 0.7	6.1 ± 0.6	6.2 ± 0.4	0.61
	5.8 [5.1, 7.1]	6.0 [5.3, 7.0]	6.1 [5.9, 7.1]	
−5 mm	6.3 ± 0.7	6.7 ± 0.7	6.4 ± 0.2	0.34
	6.2 [5.4, 7.5]	6.7 [5.5, 7.6]	6.4 [6.1, 6.9]	
−6 mm	6.5 ± 1.0	6.8 ± 0.7	6.8 ± 0.2	0.49
	6.9 [4.4, 7.2]	7.2 [5.7, 7.7]	6.8 [6.4, 7.1]	

### Histologic and histomorphometric outcomes

#### Augmented area and regenerated area

In the 8-week healing group, there were no significant differences in the augmented and regenerated areas among the 3 groups at the coronal, middle, and apical regions. However, in the 16-week group, the flexible membrane group exhibited a significantly larger augmented area at the coronal region compared to the control and other groups (2.4 ± 1.2 mm^2^, 4.3 ± 1.7 mm^2^, and 2.8 ± 0.5 mm^2^ for the control, flex group, and stiff group, respectively; *P* = 0.03) (Table S2).

In the 16-week healing group, the flexible membrane exhibited a significantly larger regenerated area in the coronal region (1.9 ± 1.2 mm^2^ for the control, 4.1 ± 1.7 mm^2^ for the flex group, and 2.7 ± 0.5 mm^2^ for the stiff group; *P* = 0.03) and in the middle region (2.4 ± 1.0 mm^2^ for the control, 4.5 ± 1.4 mm^2^ for the flex group, and 3.5 ± 0.4 mm^2^ for the stiff group; *P* = 0.01). In the apical region, both the flexible and stiff membranes demonstrated significantly larger regenerated areas (2.0 ± 0.9 mm^2^ for the control, 4.2 ± 1.0 mm^2^ for the flex group, and 3.8 ± 0.4 mm^2^ for the stiff group; *P* < 0.01) (Table 2).

**Table 2.** Histomorphometric comparison of regenerated area at 3 parts of defects depending on vertical location. Values are presented as mean ± standard deviation and median [min, max]. The Kruskal–Wallis test was performed for statistical analysis (^a^,^b^*P* < 0.05). Control: bone substitutes. Flex group: bone substitutes+ flexible collagen membrane. Stiff group: bone substitutes+ stiff collagen membrane.

Depth	Control (mm^2^)	Flex group (mm^2^)	Stiff group (mm^2^)	*P* value
8 weeks				
Coronal	3.6 ± 0.9	3.7 ± 1.7	3.3 ± 1.6	0.63
	3.7 [2.3, 4.7]	3.5 [1.7, 6.8]	2.8 [1.6, 6.4]	
Middle	3.0 ± 2.1	2.8 ± 1.9	3.2 ± 2.2	0.88
	2.5 [0.9, 6.1]	1.6 [1.5, 5.6]	2.4 [0.8, 7.3]	
Apical	2.3 ± 1.6	3.5 ± 2.0	3.3 ± 2.1	0.64
	2.0 [0.6, 5.2]	3.3 [0.8, 6.4]	3.0 [0.5, 6.9]	
16 weeks				
Coronal	1.9 ± 1.2	4.1 ± 1.7 ^a^	2.7 ± 0.5	0.03
	1.6 [0.7, 4.4]	3.8 [2.3, 7.4]	2.6 [1.8, 3.5]	
Middle	2.4 ± 1.0	4.5 ± 1.4 ^a^	3.5 ± 0.4	0.01
	2.5 [1.0, 3.9]	4.0 [3.3, 7.5]	3.4 [3.0, 4.3]	
Apical	2.0 ± 0.9	4.2 ± 1.0 ^a^	3.8 ± 0.4 ^b^	<0.01
	1.8 [0.7, 3.2]	4.0 [3.1, 5.8]	3.8 [3.3, 4.5]	

^a^ *P* < 0.05, significant difference between the control and flex group.

^b^ *P* < 0.05, significant difference between the control and stiff group.

**Fig. 6.**
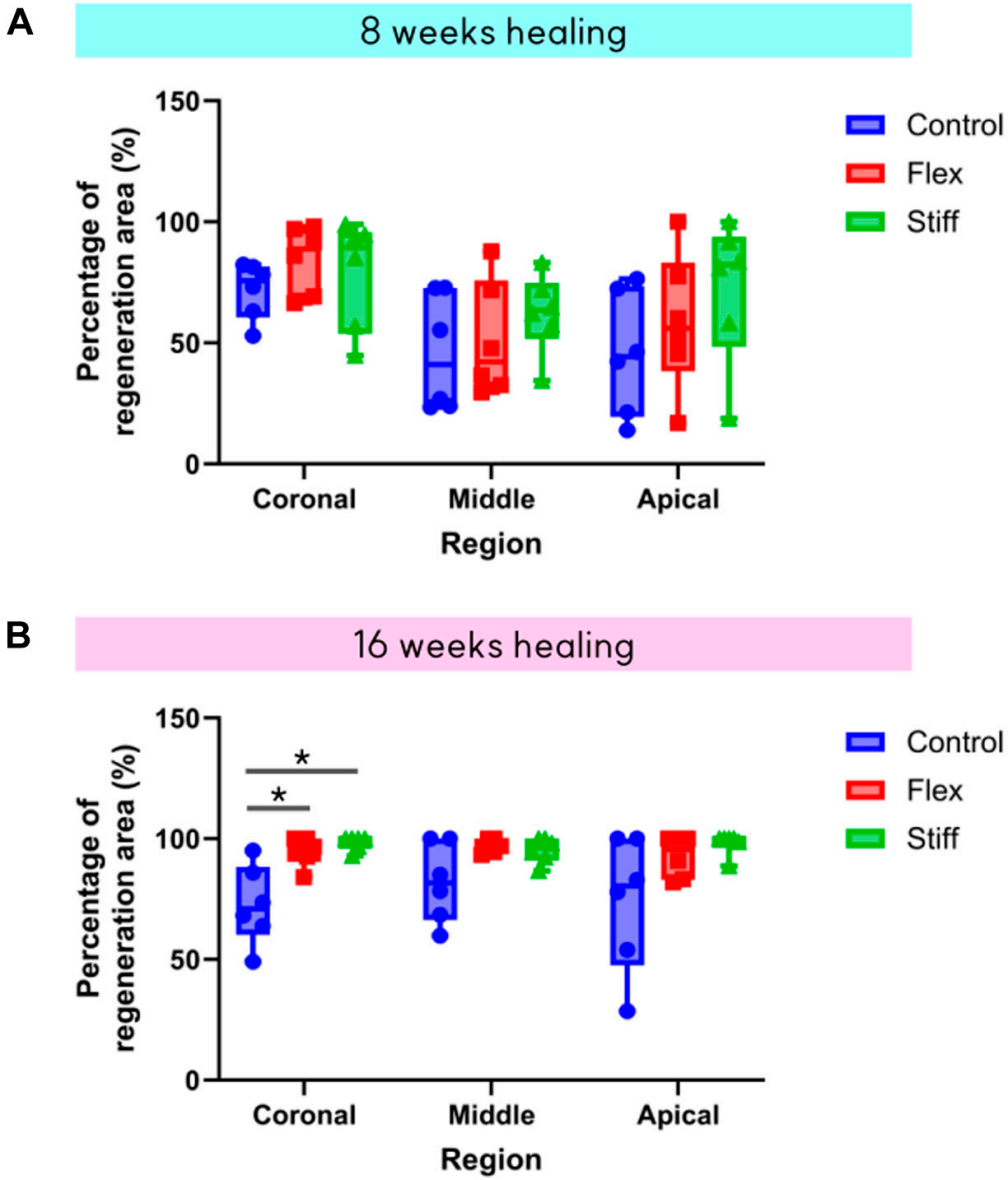
Regeneration ratios in coronal, middle, and apical areas. (A) Regeneration ratio of the 8-week healing group. (B) Regeneration ratio of the 16-week healing group. There was a significantly higher regeneration ratio in groups A and B than in the control group in the coronal region. The Kruskal–Wallis test with post-hoc test was performed for statistical analysis (^*^P < 0.05). Control, bone substitutes only; Flex, bone substitutes and flexible membrane; Stiff group, bone substitutes and stiff membrane.

All membrane groups exhibited a higher regeneration ratio compared to the control in each region. However, a significantly higher regeneration ratio was observed only in the coronal area (76.4% ± 10.4% for the control, 95.6% ± 5.4% for the flex group, and 98.2% ± 2.9% for the stiff group; *P* < 0.01) (Fig. 6).

#### Qualitative results in the regenerated area

**Fig. 7.**
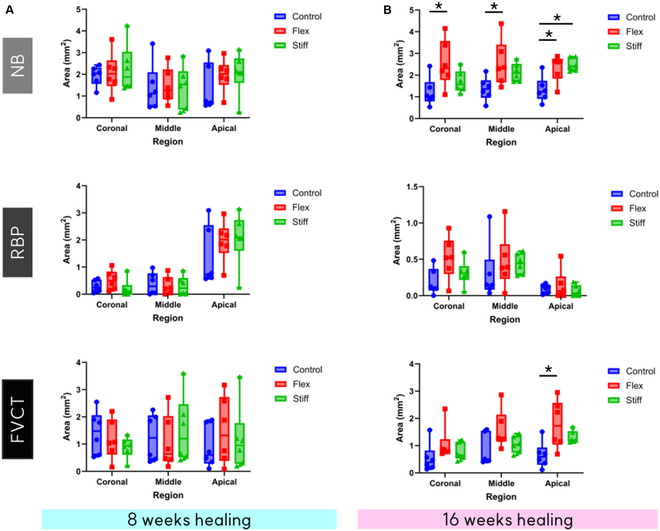
NB, RBP, and FVCT area comparison in each area (coronal, middle, and apical). (A) Eight-week healing group. (B) Sixteen-week healing group. NB, new bone; RBP, residual bone particle; FVCT, fibrovascular connective tissue. The Kruskal–Wallis test with post-hoc test was performed for statistical analysis (^*^P < 0.05). Control, bone substitutes only; Flex, bone substitutes and flexible membrane; Stiff group, bone substitutes and stiff membrane.

**Fig. 8.**
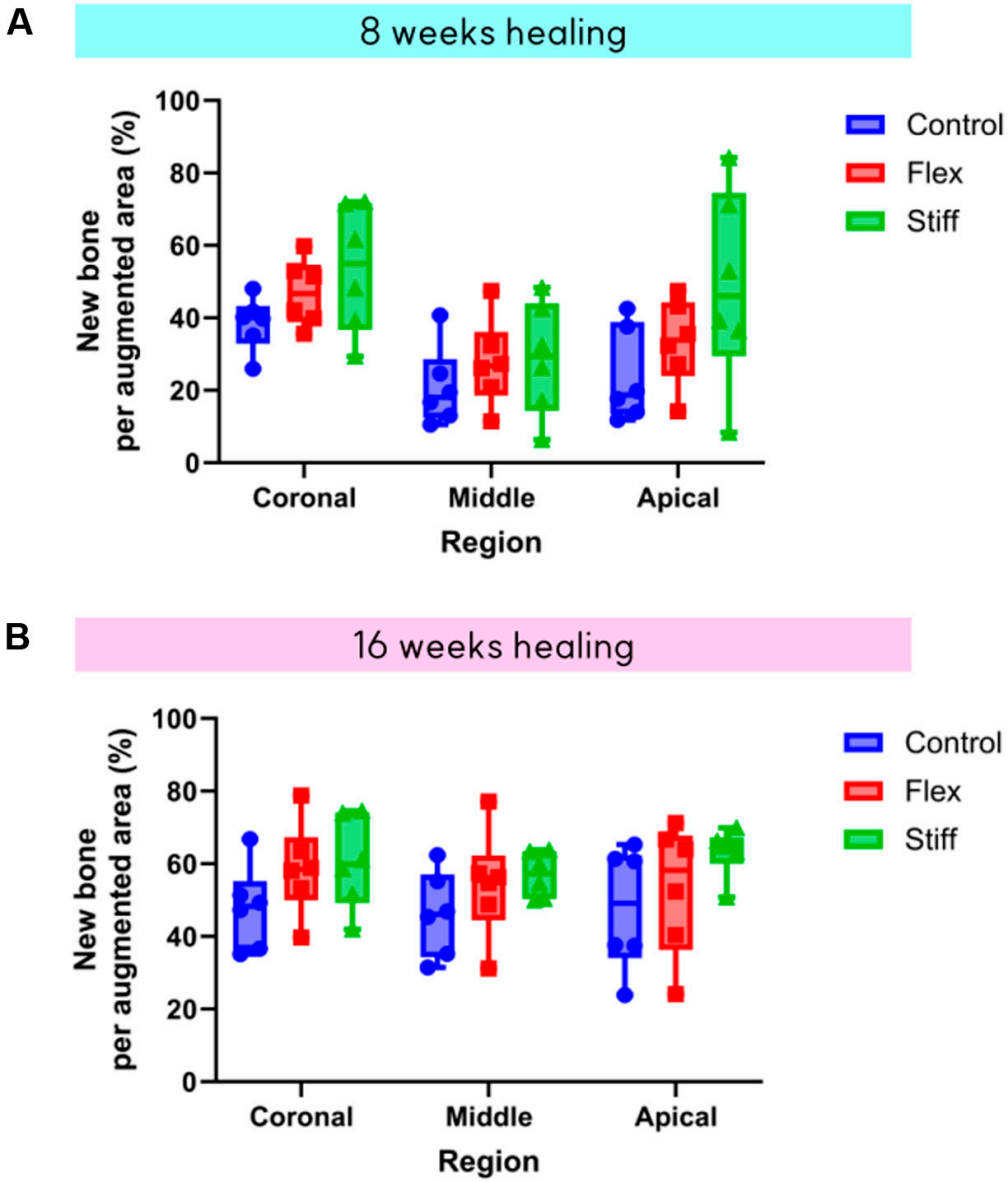
Comparison of new bone area (%) per augmented area in 8 and 16 weeks of healing at each area (coronal, middle, and apical). (A) Eight weeks. (B) Sixteen weeks. The Kruskal–Wallis test was performed for statistical analysis (^*^P < 0.05). Control, bone substitutes only; Flex, bone substitutes+ flexible membrane; Stiff group, bone substitutes+ stiff membrane.

NB, RBP, and FVCT did not show any differences among groups in the coronal, middle, and apical regions after 8 weeks of healing. At 16 weeks, the flexible membrane demonstrated significantly higher NB formation in the coronal regions (1.2 ± 0.6 mm^2^, 2.5 ± 1.0 mm^2^, and 1.7 ± 0.5 mm^2^ for the control, flex group, and stiff group, respectively, *P* = 0.03), middle regions (1.3 ± 0.5 mm^2^, 2.5 ± 1.0 mm^2^, and 2.1 ± 0.4 mm^2^ for the control, flex group, and stiff group, respectively, *P* = 0.03), and apical areas (1.3 ± 0.5 mm^2^, 2.3 ± 0.6 mm^2^, and 2.4 ± 0.2 mm^2^ for the control, flex group, and stiff group, respectively, *P* = 0.05) compared to the control. The stiff membrane also resulted in significantly higher NB formation in the apical area compared to the control (*P* = 0.03) (Fig. 7). However, there was no statistical significance between the 0.3-mm membrane vs. 0.5-mm collagen membrane for both new bone area demonstrated in Fig. 7 and new bone percent per augmented area as shown in Fig. 8.

## Discussion

The present study aimed to investigate the mechanical properties of stiff collagen membranes compared to flexible collagen membranes through in vitro tests and through radiographic and histomorphometric analyses in vivo. The flexible membrane exhibited higher hydrophilicity, underwent faster enzymatic degradation, and demonstrated greater conformability than the stiff membrane. Our findings indicated that the use of bone substitutes, including DPBM-C and DPBM, increased the alveolar ridge width in micro-CT analysis, regardless of the use of a barrier membrane. Additionally, GBR using the flexible collagen membrane significantly improved bone regeneration compared to the stiff membrane, particularly during the 16-week healing period, as shown in histomorphometric analysis.

The in vitro experiments demonstrated that the flexible collagen membrane exhibited superior hydrophilicity, faster enzymatic degradation, and greater conformability compared to the stiff membrane. These properties are important as they facilitate better integration and adaptation of the membrane with the surrounding tissues, promoting a more favorable environment for bone regeneration. Additionally, the high biocompatibility of the collagen membrane underscores its potential clinical benefits. Furthermore, the flexible membrane significantly improved bone regeneration and integration in vivo, highlighting its effectiveness in maintaining the necessary space for new bone growth and reducing micromovements that can disrupt the healing process. These findings suggest that incorporating flexible membranes into GBR procedures might improve clinical outcomes [27].

The alveolar ridge width at each depth, as analyzed by micro-CT, showed similar results across the control, flexible membrane, and stiff membrane groups. This suggests that bone substitutes, including DPBM-C and DPBM, positively influence lateral bone augmentation when used as scaffolds. Similar findings have been reported where augmentation with bone substitutes in alveolar bone defects demonstrated their ability to counteract ridge width shrinkage in micro-CT analysis [28–31]. In addition to the quantitative analysis performed using micro-CT, no differences were observed among the 3 groups in qualitative metrics such as BV/TV, BS/BV, Tb.Pf, SMI, Tb.Th, Tb.N, and Tb.Sp. These results may be attributed to the insufficient resolution of micro-CT to effectively distinguish differences within the greyscale used in this study.

All barrier membrane groups, both using flexible and stiff membranes, exhibited a higher regeneration ratio compared to the control in each region. The significant difference in bone regeneration ratios underscores the importance of mechanical properties in barrier membranes. This finding is consistent with previous studies that have highlighted the role of membranes in bone regeneration [32,33].

The superior performance of the flexible membrane in the coronal and middle regions at 16 weeks suggests that its flexibility may enhance integration with surrounding tissues. This improved integration could stem from the flexible membrane’s enhanced conformability, which ensures closer contact with both the bone substitutes and existing bone structures. This close contact helps reduce micromovements that can disrupt the healing process. Additionally, the higher bone regeneration ratios observed with the flexible membrane suggest that its flexibility helps maintain the necessary space for new bone growth. This aspect is crucial during the early stages of healing, when the bone graft is most vulnerable to collapse under the pressure from overlying soft tissues. In these initial stages post-surgery, tissue swelling can occur, and such edema can exert pressure on the bone substitutes [34], particularly when a thick and stiff membrane is used.

Moreover, the histomorphometric analysis revealed that the group using the flexible membrane exhibited significantly higher new bone formation at various vertical locations. This suggests that the advantages of membrane flexibility are widespread, affecting the entire defect site rather than being limited to specific areas. The increased new bone formation observed in the group with the flexible membrane indicates that this type of membrane may facilitate a more uniform and extensive bone regeneration process. Commercially available cross-linked collagen membrane thickness ranges from 0.2 to 0.3 mm [3,35]. In this study, the thicknesses of the flexible and stiff membranes were 0.3 and 0.5 mm, respectively. This difference in physical thickness might contribute to the observed outcomes at the GBR site. However, a previous study comparing thin (0.2 to 0.4 mm) and thick (1 to 2 mm) collagen membranes in horizontal GBR showed no significant differences in bone regeneration at 4, 12, and 24 weeks [36]. This finding suggests that membrane flexibility has a greater impact on the bone regeneration site than the membrane’s thickness. Therefore, while both types of membranes meet the basic functional requirements of a GBR membrane, flexibility appears to offer additional regenerative benefits.

The findings from this study have additional clinical implications. The use of flexible collagen membranes may be particularly beneficial in cases requiring extensive bone augmentation where maintaining the stability of the grafted material poses a challenge. By eliminating the need for additional space-maintaining structures through the use of soft block bone, the flexible membrane could simplify surgical procedures and improve patient comfort and recovery. Previous studies have reported that particle-type bone substitutes show higher new bone formation at 3 months compared to soft block-type substitutes [37]. However, this difference was not evident at 6 months. This outcome is likely due to the collagen in the soft block type, which may block the spaces needed for the infiltration of various osteogenic cells during the early stages of bone regeneration. Additionally, soft block-type bone substitutes tend to be absorbed more extensively in vivo than the particle type [37]. Therefore, a bone grafting technique that uses a soft block bone substitute to support the lower region and layers the upper region with a particulate type can be clinically significant. This method not only ensures adequate space maintenance but also minimizes the reduction of the grafted site.

Furthermore, the animal model employed in this study is a well-established tool for GBR and dental implant research and is considered a reliable proxy for human clinical studies [37–41]. Nonetheless, further clinical research is necessary to verify these findings in human patients and to assess the long-term stability and integration of the regenerated bone. Additionally, this study did not examine the soft tissue volume of the alveolar ridge. Since the stiff membrane was thicker than the flexible membrane, it could influence changes in the soft tissue. The potential for soft tissue substitutes may be explored in future studies.

In conclusion, our study demonstrates that membrane flexibility remarkably improves the outcomes of lateral bone augmentation, promoting increased bone regeneration and improved integration with existing tissues. These findings indicate that incorporating flexible membranes into GBR procedures could enhance clinical outcomes and patient experiences. Future research should focus on validating these results in clinical settings and investigating the potential of flexible membranes in other bone regeneration applications.

## Data Availability

The data that support the findings of this study are available from the corresponding authors upon request.
