# Supplementary material for: A Flexible Membrane May Improve Bone Regeneration by Increasing Hydrophilicity and Conformability in Lateral Bone Augmentation
Source: Biomater Res. 2024 Nov 18;28:0113. doi: 10.34133/bmr.0113 (PMC11570787; doi:10.34133/bmr.0113)
Supplement: Supplementary 1 — Figs. S1 and S2 Tables S1 and S2 [file bmr.0113.f1.zip › Supplementary Figure 1(revised).docx]

**
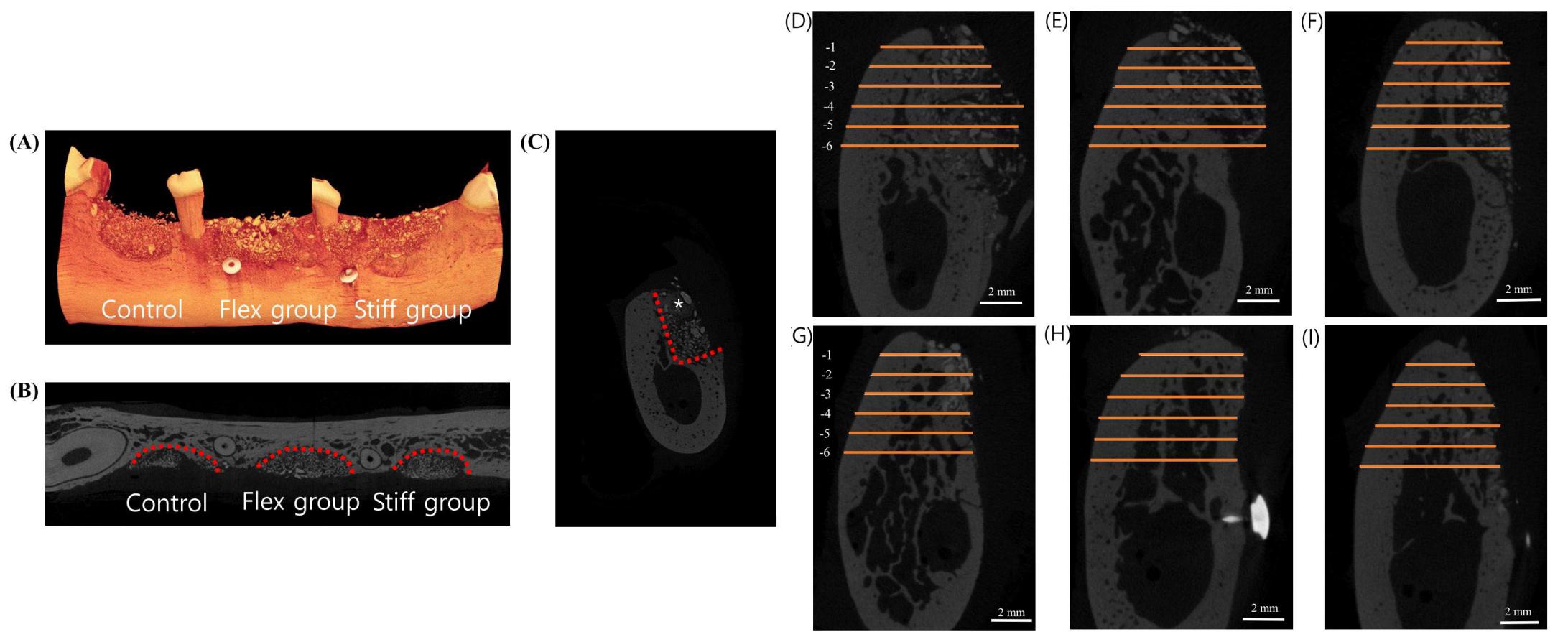
**

**Supplementary Figure 1. Representative cone-beam computed tomography images.** (A) Coronal view (B) Axial view. Augmented bone substitutes are demarcated by a red dotted line (C) Sagittal view. Grafted bone substitutes area marked with a white asterisk. (D-I) Overall width of alveolar bone measurement in 1-mm increments from 1 mm to 6 mm of alveolar crest in the apical direction. (D-F) Representative figures of the control, flex group, and stiff group, respectively, at 8 weeks of healing. (G-I) Representative figures of the control, flex group, and stiff group, respectively, at 16 weeks of healing.
