# Supplementary material for: A Flexible Membrane May Improve Bone Regeneration by Increasing Hydrophilicity and Conformability in Lateral Bone Augmentation
Source: Biomater Res. 2024 Nov 18;28:0113. doi: 10.34133/bmr.0113 (PMC11570787; doi:10.34133/bmr.0113)
Supplement: Supplementary 1 — Figs. S1 and S2 Tables S1 and S2 [file bmr.0113.f1.zip › Supplementary Figure 2(revised).docx]

**
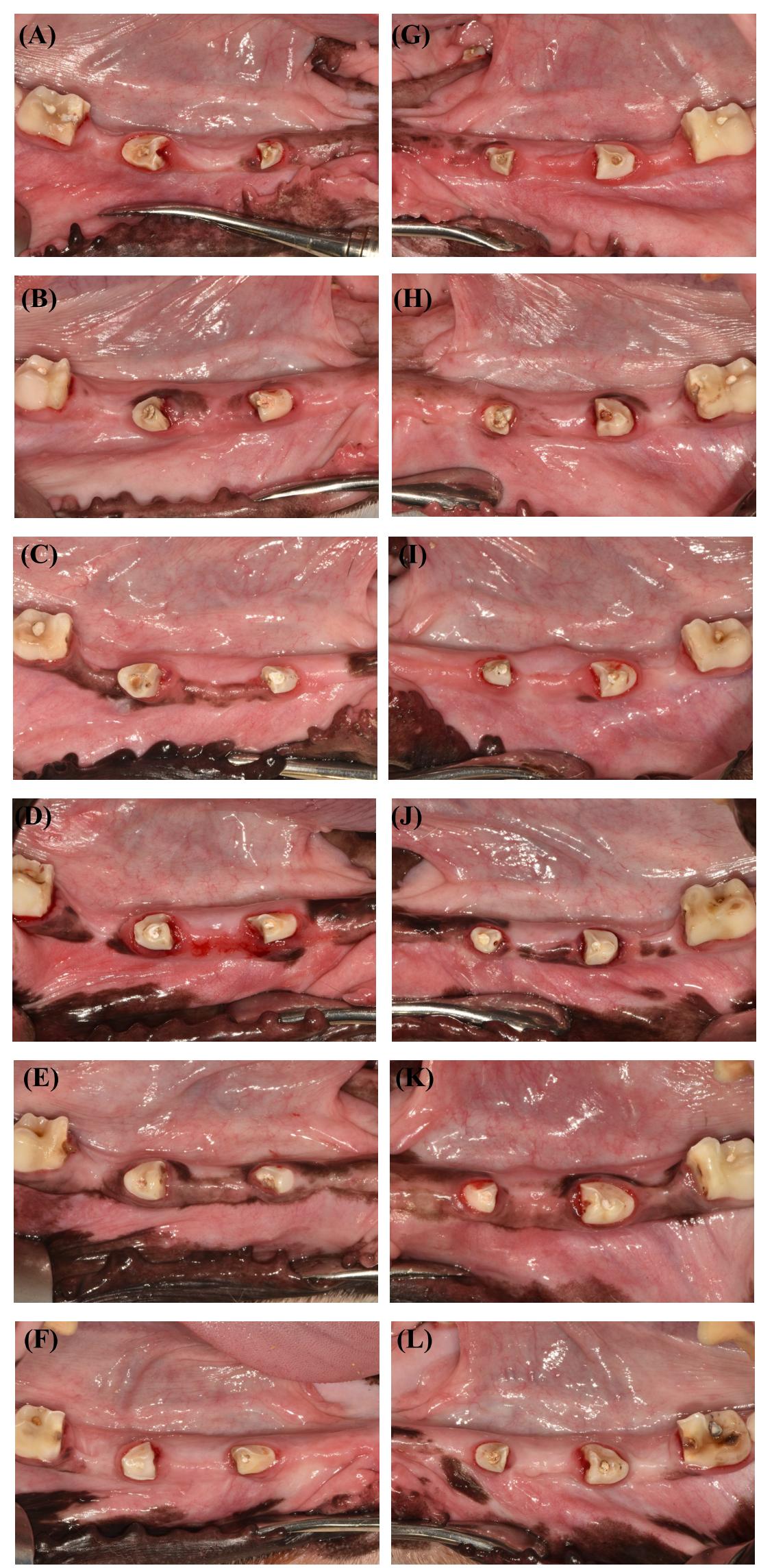
**

**Supplementary Figure 2.** (A, C, E, G and I) Right experimental sites of six beagle dogs in 8 weeks after surgical procedure. (B, D, F, H and J) Opposite experimental sites of same animals in 8 weeks after surgical procedure.
