# Supplementary material for: A Flexible Membrane May Improve Bone Regeneration by Increasing Hydrophilicity and Conformability in Lateral Bone Augmentation
Source: Biomater Res. 2024 Nov 18;28:0113. doi: 10.34133/bmr.0113 (PMC11570787; doi:10.34133/bmr.0113)
Supplement: Supplementary 1 — Figs. S1 and S2 Tables S1 and S2 [file bmr.0113.f1.zip › Supplementary Table 1(revised).docx]

Supplementary Table 1. Volumetric analysis with micro-CT comparing three groups

|  |  | 8 weeks | P-value | 16 weeks | P-value |
| --- | --- | --- | --- | --- | --- |
| Total volume | Control  Flex group  Stiff group | 436.5 ± 114.8  415.3 [327.4, 646.0]  420.0 ± 61.9  395.3 [352.3, 507.1]  424.7 ± 94.8  424.7 [301.2, 551.4] | 0.94 | 350.4 ± 42.9  358.1 [285.0, 401.3]  334.8 ± 61.4  338.3 [232.9, 400.1]  323.2 ± 48.4  331.9 [247.6, 372.4] | 0.71 |
| Bone volume /Total volume | Control  Flex group  Stiff group | 41.0 ± 5.9  40.7 [34.5, 49.3]  45.1 ± 5.0  44.3 [40.1, 51.6]  43.4 ± 6.1  42.4 [36.4, 52.6] | 0.37 | 35.5 ± 5.7  35.0 [29.7, 43.5]  41.2 ± 4.7  41.3 [35.8, 48.0]  40.0 ± 7.8  40.5 [28.2, 51.9] | 0.33 |
| Bone surface /Bone volume | Control  Flex group  Stiff group | 15.3 ± 2.8  16.5 [10.8, 18.3]  12.4 ± 1.6  13.1 [9.6, 13.8]  12.6 ± 2.9  11.9 [9.7, 18.2] | 0.19 | 15.6 ± 4.7  15.9 [9.0, 21.5]  12.7 ± 2.3  12.2 [10.0, 16.1]  14.3 ± 2.7  14.7 [11.1, 16.9] | 0.33 |
| Tb.Pf | Control  Flex group  Stiff group | -7.0 ± 1.3  -6.9 [-8.7, -5.6]  -8.7 ± 1.6  -9.0 [-10.4, -7.0]  -8.1 ± 3.4  -8.2 [-12.9, -3.2] | 0.34 | -7.0 ± 3.2  -7.5 [-10.9, -3.3]  -10.4 ± 1.7  -10.3 [-12.5, -8.0]  -8.4 ± 3.2  -9.3 [-11.5, -4.4] | 0.20 |
| SMI | Control  Flex group  Stiff group | -2.9 ± 1.1  -2.5 [-4.5, -1.8]  -4.3 ± 1.3  -3.9 [-6.4, -3.1]  -4.2 ± 2.3  -4.2 [-7.9, -1.0] | 0.19 | -3.2 ± 2.2  -3.2 [-6.2, -1.0]  -5.0 ± 1.3  -4.6 [-6.9, -3.7]  -3.8 ± 2.0  -3.8 [-6.0, -1.5] | 0.35 |
| Tb.Th | Control  Flex group  Stiff group | 0.2 ± 0.03  0.1 [0.1, 0.2]  0.2 ± 0.02  0.2 [0.2, 0.3]  0.2 ± 0.03  0.2 [0.1, 0.3] | 0.14 | 0.2 ± 0.05  0.2 [0.1, 0.3]  0.2 ± 0.03  0.2 [0.1, 0.3]  0.2 ± 0.02  0.2 [0.1, 0.3] | 0.34 |
| TB.N | Control  Flex group  Stiff group | 1.9 ± 0.2  1.9 [1.5, 2.3]  1.7 ± 0.1  1.7 [1.5, 1.9]  1.7 ± 0.3  1.8 [1.2, 2.1] | 0.22 | 1.7 ± 0.3  1.6 [1.2, 2.1]  1.7 ± 0.4  1.6 [1.4, 2.5]  1.8 ± 0.3  1.8 [1.4, 2.3] | 0.77 |
| Tb.Sp | Control  Flex group  Stiff group | 0.7 ± 0.2  0.7 [0.4, 1.0]  0.8 ± 0.2  0.9 [0.5, 1.0]  0.8 ± 0.2  0.9 [0.5, 1.1] | 0.47 | 1.0 ± 0.3  0.9 [0.7, 1.6]  1.0 ± 0.2  1.1 [0.7, 1.3]  0.9 ± 0.1  0.9 [0.6, 1.1] | 0.71 |

Values are presented as mean ± standard deviation and median [min, max]. The Kruskal-wallis test was performed for statistical analysis (^*^*P* < 0.05).

Control: bone substitutes

Flex group: bone substitutes+ flexible collagen membrane

Stiff group: bone substitutes+ stiff collagen membrane

Abbreviations: TV: total volume; BV: bone volume; BS: bone surface; Tb.Pf: trabecular bone pattern factor; SMI: structure model index; Tb.Th: trabecular thickness; TB.N: trabecular number; Tb.Sp: trabecular separation
