# Supplementary material for: A Flexible Membrane May Improve Bone Regeneration by Increasing Hydrophilicity and Conformability in Lateral Bone Augmentation
Source: Biomater Res. 2024 Nov 18;28:0113. doi: 10.34133/bmr.0113 (PMC11570787; doi:10.34133/bmr.0113)
Supplement: Supplementary 1 — Figs. S1 and S2 Tables S1 and S2 [file bmr.0113.f1.zip › Supplementary Table 2(revised).docx]

Supplementary Table 2. Histomorphometric analysis of augmented area at three parts of defects depending on vertical location (mm^2^)

| 8 weeks | | | | |
| --- | --- | --- | --- | --- |
| Depth | Control | Flex group | Stiff group | P-value |
| Coronal | 5.1 ± 1.2  5.4 [2.9, 6.5] | 4.3 ± 1.6  4.4 [2.0, 7.0] | 4.1 ± 1.4  3.6 [3.0, 6.9] | 0.33 |
| Middle | 6.0 ± 1.5  6.1 [3.8, 8.3] | 5.2 ± 1.5  5.1 [3.4, 7.8] | 4.8 ± 2.5  3.6 [2.4, 8.8] | 0.42 |
| Apical | 5.1 ± 1.5  5.1 [3.4, 7.2] | 5.9 ± 1.8  5.4 [3.7, 8.3] | 4.6 ± 2.4  3.4 [2.5, 8.6] | 0.39 |
| 16 weeks | | | | |
| Coronal | 2.4 ± 1.2  2.1 [1.0, 4.7] | 4.3 ± 1.7 ^a^  4.0 [2.7, 7.7] | 2.8 ± 0.5  2.7[1.8, 3.5] | 0.03 |
| Middle | 3.1 ± 1.3  3.2 [1.0, 4.6] | 4.7 ± 1.4  4.1 [3.5, 7.6] | 3.7 ± 0.6  3.5[3.0, 5.0] | 0.20 |
| Apical | 2.9 ± 1.0  2.9 [1.5, 4.0] | 4.6 ± 1.4  4.4 [3.1, 7.1] | 4.0 ± 0.6  3.8[3.3, 4.7] | 0.06 |

Values are presented as ± standard deviation and median [min, max]. The Kruskal-wallis test with post-hoc test was performed for statistical analysis (^a,b^*P* < 0.05).

1. Significant difference between control and flex group

Control: bone substitutes

Flex group: bone substitutes+ flexible collagen membrane

Stiff group: bone substitutes+ stiff collagen membrane
